# 한국 미혼 여성의 선택적 난자동결보존 수행 경험: 기술적 현상학적 연구

**DOI:** 10.4069/whn.2025.05.19

**Published:** 2025-09-30

**Authors:** Euna Park, Da-In Kang

**Affiliations:** 1 College of Nursing, Pukyong National University, Busan, Korea; 1 부경대학교 간호대학; 2 College of Nursing, Dong-eui Institute of Technology, Busan, Korea; 2 동의과학대학교 간호대학

**Keywords:** Cryopreservation, Oocytes, Qualitative research, Single person

## Abstract

**Purpose:**

The increasing prevalence of elective oocyte cryopreservation (EOC) for fertility preservation has generated growing interest worldwide. This study explores the experiences of unmarried women undergoing EOC.

**Methods:**

This descriptive phenomenological study sought to investigate the meaning and structure of the EOC experience among healthy unmarried women. Five unmarried women who had undergone EOC participated in the study. Data were collected through in-depth interviews and observations via an online platform. Interviews were conducted in a 1:2 format between participant and researcher, with each participant interviewed once or twice. The primary interview question was, “Please tell us about the circumstances under which you decided to undergo EOC.”

**Results:**

From 65 units of meaning, six themes and 19 subthemes emerged: an innovative tool that expands life choices; a process that requires readjusting life priorities; a conflict felt at the boundary between tradition and change; fierce information search and perseverance amidst the turmoil of body and mind; intertwined with possibilities, constraints, and expectations for the future; and feeling uncertain about freedom of choice and taking initiative for the future.

**Conclusion:**

Unmarried women have experienced various difficulties in the process of EOC depending on the situation related to the passage of time. They need to have accurate information to perform this, and there needs to be discussions on how to use or dispose of the oocytes when they are not in use. In addition, in order to solve the problem of low birth rates, there is a need to increase the cost support for EOC.

## Introduction

난자동결보존(oocyte cryopreservation)은 향후 잠재적인 사용 가능성을 열어 두기 위해 난자를 동결하여 생식능력을 보존하는 데 목적을 둔 보조생식기술(assisted reproductive technology)이다[1]. 1986년에 이전에 동결보존해 두었던 난자를 이용한 최초의 쌍둥이 임신이 성공한 이래로[2] 난자동결보존 기술이 급격히 향상되어 난자의 생존율과 임신율이 크게 증가하였다[3]. 난자동결보존은 주로 의료적 목적 혹은 선택적 목적으로 수행하게 되는데, 2014년 대부분의 난자동결보존은 항암화학요법 및 골반 방사선을 포함한 성선 독성요법에 노출되는 환자나 원발성 난소기능부전을 유발하는 유전적 장애가 있는 환자 등 의료적 목적으로 사용되었다[4]. 2018년 미국 생식의학회에서 생식노화로 인한 불임 가능성이 있는 사람에게도 난자동결보존을 허용하면서 전 세계적으로 선택적 목적의 난자동결보존이 증가하게 되었다[5].

지금까지 난자동결보존은 사회적 난자동결(social egg freezing), 비의학적 난자동결(nonmedical egg freezing), 선택적 난자동결보존(elective oocyte cryopreservation), 선택적 생식능력 보존(elective fertility preservation), 예상되는 생식능력 고갈을 위한 난자은행(oocyte banking for anticipated gamete exhaustion) 등의 용어를 혼용하여 사용하고 있으나[6,7] 2018년 호주 및 뉴질랜드 생식 내분비학 및 불임 협회(Australian and New Zealand Society of Reproductive Endocrinology and Infertility)에서는 사회적 난자동결보존이라는 용어는 여성에게 사회적 낙인이나 비난을 초래할 가능성이 있으므로 선택적 혹은 계획적 난자동결보존이라는 중립적이고 비판단적 용어를 쓸 것을 권고하고 있다[8].

역학연구에 따르면 선택적 난자동결을 선택하는 여성은 일반적으로 36세에서 40세 사이의 백인이며, 고등교육을 받았고, 전문 직업을 가지고 있으며, 파트너가 없는 것으로 나타났다[9,10]. 다른 연구에서는 여성들이 주로 교육이나 경력 향상 등의 목적으로 생식능력을 지연 또는 연기하여 생식 자율성을 달성하고[11,12] 연령 관련 생식능력의 감소를 줄이기 위해 난자동결보존을 시행하는 것으로 나타났다[13-15]. 그러나 가임기 여성들은 여전히 연령 증가가 가임력에 미치는 영향을 충분히 인식하지 못하고 있고, 난자동결보존에 대한 정보를 의료인이 아니라 온라인에서 얻는 경우가 많았다[16]. 또한 의료인들은 생식 노화에 대한 다양한 측면의 논의가 있어야 한다고 생각은 하지만 대상자를 상담할 시간이나 지식이 부족하다고 보고하고 있어[17], 대상자와 의료인들이 난자동결보존에 대한 정확한 정보와 인식이 부족한 것으로 나타났다. 난자동결보존을 한 영국, 미국, 노르웨이에 거주하는 31명의 참여자를 대상으로 한 질적 연구에서[18] 여성들은 이를 수행한 동기가 파트너를 찾을 시간과 자연 임신을 할 시간이 부족하다는 느낌, 미래에 난자를 동결보존해 놓지 않은 것에 대한 후회와 비난에 대한 두려움, 이를 결심하게 된 주변의 여러 가지 사건의 존재 등을 언급하면서, 이러한 결심이 단순히 모성이 되는 시간을 지연시키기 위함은 아니라고 밝혔다.

국내 난자동결보존에 관한 선행연구를 살펴보면, 여성 암 환자의 가임력 보존에 대한 인식 연구[19], 가임력 보존을 위한 난자 냉동보존에 관한 연구[20,21] 등이 있었다. 그러나 난자동결보존에 대한 국내 선행 연구들을 살펴보면 대부분이 문헌고찰 연구이거나 환자를 대상으로 한 연구로서, 건강한 미혼 여성의 난자동결보존 경험을 이들만의 관점에서 전체적으로 깊이 있게 이해하는 데에는 한계가 있다.

미혼 여성의 선택적 난자동결보존 경험을 이해하기 위해서는 이들의 생생한 진술을 통해 이들의 경험을 드러내고, 나아가 이들이 그 경험에 어떤 의미를 부여하고 있는가를 규명할 수 있는 연구가 필요하다. 이에 본 연구에서는 Giorgi [22]의 기술적 현상학적 연구 방법을 통해 미혼 여성의 선택적 난자동결보존 과정에서 경험하는 감정과 선택적 난자동결보존의 의미를 드러내고 상황적 맥락에 따른 개인 경험의 본질적 구조를 파악함으로써 선택적 난자동결보존 경험에 대한 이해를 높이고자 한다.

## Methods

**Ethics statement**: This study was approved by the Institutional Review Board of Pukyong National University (No. 1041386-202308-HR-78-01). Participants were given information on the study and engaging in the online interview was considered as consenting to the study.

### 연구 설계

본 연구는 미혼 여성의 선택적 난자동결보존 경험의 의미와 구조를 탐구하기 위해 Giorgi [22]의 기술적 현상학적 연구 방법을 적용한 질적 연구이다. 본 연구는 SRQR (Standards for Reporting Qualitative Research) 보고지침(https://www.equator-network.org/reporting-guidelines/srqr)을 준수하여 기술하였다.

### 연구 참여자

본 연구의 참여자 선정 기준은 15세에서 49세 사이의 가임기 미혼 한국인 여성으로, 첫 번째 난자동결보존을 시행한지 1년 이내인 자로 하였다. 제외 기준은 외국에서 난자동결보존을 시행한 경우이다. 참여자는 목적적 표집 방법과 눈덩이 표집 방법에 따라 선정하였다. 연구 참여자 모집을 위해 모집 공고문을 인스타그램, 페이스북 등과 같은 소셜 네트워크 서비스(SNS)에 게시하여 참여 의사를 받았으며, 또한 포털 사이트 블로그에서 ‘난자동결’ 혹은 ‘난자냉동’ 등 관련 키워드를 작성한 사용자에게 개별 메시지로 연구 모집 공고문을 전달하였다.

### 연구자 준비

본 연구에서 제1저자는 난임 부부의 보조생식술 실패 경험을 연구하는 과정에서 미혼 여성의 난자동결보존에 대해 관심을 가지게 되었다. 저자는 질적 연구 설계, 질적 내용 분석, 현상학, 근거 이론 등 질적 연구 방법론을 꾸준히 익혀왔으며, 질적 연구 특강과 워크숍 등에 참여해 해당 역량을 지속적으로 개발해 왔다. 또한, 박사과정에서 질적 연구 방법론을 강의하고 있으며, 질적 연구 방법론을 이용한 연구를 학회지에 여러 편 게재한 경험이 있다. 교신저자는 대학원 박사과정에서 질적 연구 방법론을 수강하였으며, 질적 연구를 수행하는 학회에 소속되어 관련된 특강에 참여하면서 질적 연구 수행을 위한 역량을 쌓았다. 연구자들은 이론적 민감성을 유지하기 위해 연구 자료를 반복적으로 검토하며 중요한 의미를 도출해 내기 위해 노력하였다.

### 자료 수집

본 연구의 자료 수집은 2023년 10월부터 2024년 3월까지 약 5개월간, 참여자와 연구자 간 1:1 심층면담과 관찰을 통해 이루어졌다. 연구자 1인은 녹화에 대한 동의를 구하고 주요 면담을 진행하였으며, 연구 보조자는 참여자의 표정, 어조, 동작 등의 비언어적 표현을 관찰하고 메모하였다. 참여자별 면담 횟수는 1–2회였으며(2회 면담 시행: P1, P2, P3), 1회 면담의 평균 소요 시간은 64분(50–80분)이었다. 2차 면담은 전화 및 비대면으로 진행되었으며 약 30분이 소요되었다.

면담은 참여자가 수도권 또는 국외에 거주하고 있어, 참여자의 편의를 고려해 Zoom을 이용하여 진행되었다. 면담 시 참여자가 자신의 경험을 자유롭게 이야기할 수 있도록 주요 질문과 후속 질문을 사용하였다(Table 1). 자료 수집은 참여자와의 면담 자료를 분석하면서 연구자 간 자료의 포화에 이르렀다고 판단될 때까지 진행되었다. 연구 참여자의 동의를 얻어 면담을 녹화하였으며, 면담 종료 후 당일 녹화된 내용을 들으며 참여자가 표현한 언어를 그대로 필사하여 녹취록을 작성하였다. 면담이 끝난 후, 참여자들에게는 소정의 모바일 쿠폰(3만 원 상당)을 지급하였다.

### 자료 분석

자료 분석은 자료 수집과 동시에 이루어졌으며, 반복적인 순환과정을 거쳤다. 미혼 여성의 난자동결보존 수행 경험의 의미와 본질 구조를 파악하기 위해 Giorgi [22]가 제시한 기술적 현상학적 연구 방법에 따라 전체적 인식, 의미 단위 분석, 의미 단위의 학문적 용어로의 변형과 구조로의 통합이라는 4단계 분석을 통하여 참여자의 진술에 중점을 두어 체험 자체의 의미를 파악하고, 개별적 체험 들에서 공통된 일반적 의미를 발견할 수 있도록 하였다. 첫째, 연구자들은 녹음 내용을 필사한 자료를 독립적으로 처음부터 끝까지 여러 차례 읽으면서 참여자들 경험의 총체적 의미와 맥락을 파악하였다. 즉, 참여자별로 선택적 난자동결수행 경험이 어떤 것인지에 대해 전체적인 모습과 흐름을 확인하였다. 둘째, 연구 주제 현상에 초점을 맞춰 중첩되는 의미 단위들을 하나의 의미 단위로 변경하고 의미의 전환이 일어나는 부분을 중심으로 의미 단위를 구분하여 연구 참여자의 언어로 표현된 의미 단위들을 확보하였다. 이때 내용이 불분명하거나 명확하지 않은 단어나 구 등이 발견되면 참여자에게 그 의미를 다시 질문하여 해당 내용을 보완하는 과정을 거쳤다. 셋째, 확정된 의미 단위들은 다른 의미 단위 및 전체적인 의미와 연관 지으면서 이들의 의미를 해명하고 반성과 자유 변경 과정을 통해 연구 참여자의 언어로 표현된 의미 단위들을 학문적 언어 또는 일상언어로 변경하였다. 참여자들의 선택적 난자동결수행 경험에 대한 의미 단위를 간호학적 관점과 용어로 전환하여 기술하였다. 넷째, 분석 과정을 통하여 드러난 의미 단위들을 종합하면서 관련 구성 요소들을 분석 및 합성하고 재편성하여 선택적 난자동결수행이라는 현상의 본질 구조를 파악하였다. 전체 과정에서 연구자들은 독립적으로 필사 자료를 반복해서 읽고 초기 코딩을 한 후 3회 이상의 연구팀 회의를 통한 합의과정을 통해 전체 합의를 이루었다. 최종 연구 결과는 참여자 경험을 상황과 함께 기술하는 ‘상황적 구조’와, 참여자의 공통된 경험에서 도출한 일반적 의미인 ‘일반적 구조’로 구분하여 기술하였다.

### 연구의 타당성 확립

연구의 타당성을 확립하고자 Morse 등[23]이 제시한 검증(verification), 확인(validation) 및 타당도(validity)의 측면에서 검토하였다. 검증 기준은 연구 방법에서 제시한 자료 수집과 분석 절차를 따라 진행하였고, 현장일지, 메모, 연구 관련 개인일지 등을 비교하면서 기술하는 작업을 연구가 완결될 때까지 계속함으로써 충족되었다. 확인 기준은 다양한 실제 경험 자료를 확보하고, 분석 과정에서 분류 항목과 해당 자료의 의미에 대한 연구자들 간 상호 점검, 일부 연구 참여자들의 자료 점검 등을 통하여 이루어졌다. 타당도 기준을 충족하기 위해 참여자 구성을 다양하게 하고, 자료 수집 및 분석을 다단계에 걸쳐 순환적으로 실시하였으며, 질적 연구 경험이 풍부한 교수 2인으로부터 연구 결과에 대한 타당성 검토를 받았다.

## Results

본 연구 참여자 5명의 연령은 30대가 4명, 40대가 1명이고, 평균 연령은 36세였다. 교육 정도는 모두 대학원 졸업이었다. 직업은 전문직 회사원 4인, 사업 1명이었으며, 초경 연령은 초등학교 3학년에서 중학교 1학년 사이였다. 난자동결보존 시행 횟수는 모두 1회였고, 동결보존한 난자 개수는 4–30개로 평균 16.4개였다. 한국에서 선택적 난자동결보존은 모두 비급여로, 난자동결보관까지 드는 비용은 350만 원에서 500만 원 사이였는데, 이는 2024년 도시근로자 1인 가구 기준 월평균 소득인 3,598,715원[24]과 비슷한 수준이다(Table 2).

Giorgi [22] 분석 방법에 따라 참여자 진술문을 토대로 미혼 여성의 선택적 난자동결보존 경험의 총 65개의 의미 단위를 도출하였으며, 이를 기반으로 19개의 하위 주제(subtheme)와 6개의 주요 주제(theme)를 구성하였다(Tables 3, 4). 이에 따라 분석 결과를 상황적 구조와 일반적 구조로 기술하였다.

### 참여자의 상황적 구조 기술

#### 주제 1. 삶의 선택지를 넓히는 혁신적 도구

##### 1) 미래 대비를 위한 보험

참여자들은 난자동결보존을 즉시 출산을 선택하지 못하는 상황에서 시간을 벌어주는 일종의 보험처럼 여기며, 생체시계에 쫓기지 않고 더 여유롭게 미래의 가능성을 탐색하고 싶어하였다.

“출산을 바로 지금 당장 하지 못하는 경우를 위해서 좀 킵 해놓은 보험 같은 거예요.” (참여자 1)

“난자동결보존은 시간을 사는 것 같아요. 가능성을 사는 거지요. 시간을 잠시 얼려주는...” (참여자 2)”

“저의 젊음을 얼려놓는 것, 혹시라도 있을지 모를 미래는 대비하는 그런 개념이라... 약간 보험 같은 것이에요.” (참여자 4)

“나에게는 쓰고 싶지 않은 보험 같은 것이지요. 생체시계에 쫓기고 싶지 않은...” (참여자 5)

##### 2) 전통적인 결혼과 출산의 틀을 벗어난 행위

참여자들은 난자동결보존에 대한 사회적 인식이 낮고, 이에 대한 오해와 비판이 많으며, 여성들 중에서도 일부만이 이를 긍정적으로 바라본다고 하였다. 또한 대부분의 사람들은 결혼과 출산을 자연스럽게 받아들이지만 난자동결보존은 불필요하다고 생각하거나 비판적인 시각을 가지고 있다고 하였다.

“주변에서 저를 좀 별나다고...” (참여자 1)

“제가 들었던 반응 중에 제일 어이가 없었던 게 왜 결혼 안 하게? 남자 싫어? 남자 없이 혼자 애 낳고 싶어 너도? 사유리처럼.” (참여자 3)

##### 3) 여성의 신체적 자유와 선택권 확장

참여자들은 난자동결보존이 여성에게 자유와 자아실현의 도구로서 중요한 선택권을 제공하며, 이를 통해 임신 시기를 자유롭게 결정할 수 있는 옵션을 주어 여성의 삶에 긍정적인 영향을 미친다고 생각하고 있었다.

“여성을 자유롭게 했던 큰 발명품이 저는 세 가지라고 생각하거든요. 첫 번째가 콘돔, 콘돔이 없었으면 여성이 계속 임신을 해야 되잖아요. 두 번째가 자위기구, 이로 인해 여성에게 약간의 자유와 해방이 있었다고 생각해요. 세 번째가 난자동결, 나이의 압박을 최대한 줄인 상태에서 자신이 임신하고 싶은 나이를 선택할 수 있는 옵션이 주어진 거지요.” (참여자 2)

“난자동결은 그냥 자유인 것 같아요. 자유 그 자체… 그리고 자아실현의 도구.” (참여자 5)

#### 주제 2. 삶의 우선순위 재조정이 필요한 과정

##### 1) 미뤄지는 결혼

참여자들은 난자동결보존에 대한 생각을 오래 전부터 가지고 있었지만, 높은 비용과 복잡한 절차가 예상되어 결심을 쉽게 내리지 못했으며, 결혼과 출산을 미루던 중 연애 중단과 나이 증가로 인해 불안감을 느끼면서 이를 실행하게 되었다.

“일단 가격이 엄청 비싼 줄 알아서 알아보지도 않았고, 알아보기도 되게 쉽지가 않고 애매하고... 그래서 막 진짜 몇천만 원 하는 줄 알았어요… 오래 만나던 남자 친구랑 헤어지고 막 이러면서 나이도 갑자기 확 들고 이러니까 약간 겁이 났어요. 저는 벌써 35세를 넘어섰는데...” (참여자 1)

“저는 제가 되게 일찍 결혼해서 이 나이가 되면 애가 한 서넛 있을 줄 알았는데 지금 애 아빠가 없어서 애를 못 낳고 있어요. 그래 가지고 뭐라도 해야겠다 싶어서.” (참여자 3)

“여기에 대해 허들이 너무 높으니까 하기가 되게 어려운 느낌이 있었어요.” (참여자 4)

##### 2) 사회적 기대와 개인적 목표의 갈등

참여자들은 외국 유학 중 혹은 친구를 통해 난자동결보존에 대해 알게 되었고, 높은 학력과 야망을 가진 여성들이 이 기술의 주요 대상이 될 것이라는 인식을 가지고 있었지만, 자신의 직업에 대한 애착과 주변 사례를 통해 용기와 희망을 얻게 되었다고 기술하였다.

“미국 MBA를 갔을 때… 업체 그런 데서 와서 설명회를 열었어요. MBA 오는 여자들은 대부분 사회적으로 좀 능력이 좋은 고학력 애들이고, 꿈도 크고 막 야심도 크고... 일 때문에 애 안 낳는다 이런 애들이 막 모여 있으니까.” (참여자 2)

“결심에는 직업적인 면이 컸던 것 같아요. 계속 일을 하고 싶다. 그리고 세상이 원하는 시간표에 맞춰 애기를 갖거나 누군가를 만나고 싶은 생각은 없다.” (참여자 5)

##### 3) 미래 부모로서의 책임과 고민

참여자들은 난자냉동보존에서 나이가 물론 중요한 요소이지만 개인의 건강 상태나 환경적 요인도 고려해야 한다고 생각하고 있었다. 참여자들은 둘째를 가질 가능성을 열어두면서, 경력에 미치는 영향을 고려한 현실적인 선택을 내렸으며, 아이의 건강이 최우선이라는 생각을 가지고 있었다. 그 결과 미래를 준비하는 방식으로 난자동결보존을 선택하였다.

“두 개의 시각이 아직 반반이고, 사람들의 실례가 주변에 많지 않다 보니 뭐가 정말 맞는 답인지에 대해서 사실 불확실한 거지요.” (참여자 2)

“대학원 공부를 하면서 기형, 자폐 등 여러 가지를 배우면서 이런 아이를 둔 부모가 얼마나 고생하는지도 알게 되고 특히 지능장애라든지 그런 경우에 부모들이 어떤 삶을 사는지도 너무 잘 보이니까. (중략) 봉사활동 중 시설의 아동이 어디서 뭐가 잘못된 걸까? 특수아동의 이해, 아동심리치료 등을 막 배우면서 걱정이 되었지요.” (참여자 3)

“제 친한 친구가 난자동결에 대해서 벌써 한 20대 중반부터 계속 고민을 하고 저한테 그 인식을 좀 쌓아줬어요. (중략) 웬만하면 이제 가정을 만들게 된다면 다복한 가정을 만들고 싶은데... 늦게 결혼하면 둘째가 걱정이 되는 거예요.” (참여자 4)

“외국에서는, 저는 독일에서 오래 살았기 때문에 조금 일찍 접한 것 같아요. (중략) 외국에서 항뮬러 호르몬 검사를 했어요. 애기를 많이 낳고 싶어서...” (참여자 5)

#### 주제 3. 전통과 변화의 경계에서 느끼는 갈등

##### 1) 신여성, '쎈여자'로 취급받음

참여자들은 난자동결보존에 대한 사회적 인식이 부정적이라서 많은 사람들이 이를 결정하는 것을 미루지만, 자신은 이를 열린 마음으로 받아들이고 삶에 도움이 되는 선택으로 여기고 있다. 따라서 회사에도 이를 공개적으로 알리며 자신의 경험을 공유함으로써 사람들이 이를 긍정적으로 받아들일 수 있기를 바란다.

“난자동결에 관심을 가져서 액션을 한 여자들은 다 약간 신여성 끼가 있어요. 진짜 액션까지 한 애들은 약간 외국에서 살다 왔거나 국적이 외국이랑 좀 연결되어 있거나... 이미 멘탈이 약간 오픈 마인드인 애들이 많아요... 완전 토종은 잘 못해요. 아직 우리나라의 인식이 부정적이라서… (중략) 남자들은 난자동결을 했다고 하면 뭐야~ 이렇게 본다거나 쎈여자로 생각해요.” (참여자 1)

“이걸 하려면 회사 다니는 중간중간 병원에 가야 되니까 일에서 빠져야 되잖아요. 그래서 대대적으로 얘기를 다 했어요. 신기해하더라구요. 저희 회사가 남초여서 남자밖에 없거든요.” (참여자 2)

“애초에 지지해 주는 사람들 자체가 너무 극소수였어요. 그러면 저는 속으로 생각하지요. 우리나라 한참 멀었다.” (참여자 3)

##### 2) 세대 간 가치관의 갈림길에서 양가감정을 느낌

참여자의 부모는 자식의 난자동결보존 결정에 대해 이해하지 못하고, 결혼을 빨리 할 수 있을 것이라고 믿으며, 성급한 결정이라고 생각하였다. 반면 어떤 부모는 자식의 결단을 대단하다고 느끼고 많은 사람들이 생각만 하던 일을 실제로 실행한 것에 대해 놀라워하였다.

“엄마는 별로 안 좋아했어요. 뭐 그렇게까지 해야 되니?” (참여자 2)

“부모님께 이걸 하려고 한다고 얘기했을 때 부모님은 진짜 제가 이해가 안 간다고 했고, 실행한 후에도 부모님은 지지가 뭐예요… 저 혼자 다 했지요 뭐.” (참여자 3)

“부모님은 굉장히 보수적이에요. 저는 전혀 보수적이지 못하지만…그럼에도 불구하고 우리 부모님은 저를 응원해 주셨어요. 자기 딸 얘기가 되고 나면 세대가 달라도 그렇게 생각해 주시더라구요.” (참여자 4)

##### 3) 산업화된 시술과 사회적 인식 사이에서 회의감을 느낌

참여자가 전문직을 가진 친구에게 이를 실행한다고 하였을 때 난자동결보존을 여성의 불안감을 기반으로 성장하는 산업이라고 보고, 이와 같은 대처에 대해 회의적인 시각을 가지고 있었다.

“친구들이 ‘난자 얼린다고? (허허…)’ 막 이러면서… ‘정신 나갔냐? 그 돈으로 결혼을 해라.’ 굉장히 안티 반응을 보였어요.” (참여자 3)

“외국의 의사 친구들한테 이 시술에 대해 얘기했더니 이건 사실 그냥 하나의 산업일 뿐이고 여자들의 두려움을 이용해서 계속 점점 커져가는 산업일 뿐이다.” (참여자 5)

#### 주제 4. 몸과 마음의 흔들림 속에서 치열한 정보 탐색과 인내

##### 1) 임신 준비와 맞먹는 노력을 함

참여자들은 난자동결보존을 준비하면서 일정과 신체 상태의 어려움을 극복하기 위해 건강 관리와 생활 방식을 철저히 조정하며 신중하게 준비하는 과정을 거쳤다.

“바로 하려니까 또 몸을 좀 만들어야 될 것 같은 거예요. 영양제, 밥 잘 먹고... 저는 사실 다이어트를 평소에 좀 많이 하거든요, 그래 가지고 사실 그게 좀 걱정이 되었어요. 한 6개월 정도 몸을 클린하게 만들어서 시작했어요.” (참여자 1)

“난자 채취 전에 제가 술 마시는 걸 좋아하는 편인데 6개월 동안 술을 입에도 안 대고, 그리고 그냥 평소에 제가 요가하고 운동하고 그리고 영양제를 좀 먹었어요. 엽산 같은 거.” (참여자 5)

##### 2) 다양한 매체를 활용한 병원 선택에 힘씀

참여자들은 지인 추천과 다양한 정보원을 바탕으로 신뢰할 수 있는 병원을 선택하여 난자동결보존을 실행하였다.

“지인 추천을 받아서 선생님을 딱 찍어서 병원을 갔어요.” (참여자 1)

“지인의 추천과 인터넷으로 저도 커뮤니티 같은 데다가 검색도 하고, 칼럼 기사, 여성 잡지 등을 참고하여 언급된 병원에서 했어요.” (참여자 2)

##### 3) 호르몬 주사의 부작용으로 인한 정서적 기복과 심리적 어려움을 버텨냄

참여자들은 난자동결보존을 준비하면서 감정의 출렁임을 느꼈다. 특히 과배란 유도와 주사 과정에서 힘들었고, 이는 주변 사람들과의 관계에도 영향을 미쳤다.

“과배란을 유도하는 과정에서 일주일 동안은 사실 정서적으로 우울하고 힘들기는 해가지고...약간 현타가 오긴 해요.” (참여자 1)

“주사 맞는 동안 막 힘들진 않았는데 상사한테 짜증을 엄청 부렸더니 상사가 ‘야, 너 주사 맞는 거 거기 호르몬 같은 것도 들어가냐?’고(웃음).” (참여자 2)

“준비 과정에서 주사를 맞는 동안 제가 원래는 되게 이성적인 사람인데, 감정 기복이 되게 심했고, 슬픈 느낌이 많이 들었어요.” (참여자 4)

“정신적으로 주사를 맞을 때의 그 약간의 걱정과 약간 자괴감도 들었던 것 같아요. 뭐 얼마나 원한다고 이렇게까지 하지?” (참여자 5)

##### 4) 신체적 변화와 회복의 더딤을 수반하는 도전을 견딤

참여자들은 난자 채취 전과 후에 신체적 어려움을 겪었으며, 주사와 과배란 유도로 인한 부종, 피로, 통증 등과 함께 복수, 변비, 멀미 등의 부작용으로 인해 일상생활에도 어려움을 겪었다. 이러한 경험을 통해 이 과정이 예상보다 훨씬 더 힘든 과정임을 깨달았고, 임신과 출산에 대한 이해가 깊어졌다.

”몸이 엄청 무겁고 아랫배가 묵직하고 생리통 비슷한 증상이.” (참여자 1)

“채취 후가 진짜 힘들었어요. 제가 워낙 채취 수가 많다 보니까 채취를 많이 하면 그만큼 복수가 찰 위험이 크다고 하더라고요. 그래서 복수 안 차게 하기 위한 약을 먹었더니 온몸에 있는 수분이 다 말라서 변비가 엄청 크게 온 거예요. 변비 때문에 지옥을 경험했지요.” (참여자 2)

“채취 당일부터 몸이 좀 완전히 돌아온 것 같다, 까지는 한 2, 3개월이 걸렸던 것 같아요. 채취 후에는 진짜 애 낳은 사람처럼 몸도 막 불고 얼굴도 커지고 가슴도 변하고... (중략) 이게 진짜 보통 일이 아니던데요? 저는 진짜 거의 반 죽었거든요. 그냥 느낌으로는 유산 한 번 한 정도의 데미지…아예 활동이 불가능할 정도였어요.” (참여자 3)

“신체적으로는 몸이 많이 붓죠. 첫 번째, 두 번째 날은 머리가 많이 아프고 엄청 피곤하고 거의 계속 잤던 것 같아요.” (참여자 4)

“멀미가 원래 없는데 그 약을 먹는 동안 약간 미식거리고 막 어지럽고, 변비도 심하고 해서 솔직히 회사 일 하는 게 좀 많이 힘들었어요.” (참여자 5)

#### 주제 5. 가능성과 제약, 미래를 향한 기대가 얽힘

##### 1) 중장기 유지 계획을 세움

참여자는 난자동결보존을 적어도 40대 후반까지는 지속할 계획이며, 보관비용이 크게 부담되지는 않는다고 하였다. 병원에서는 보관기한이 사실상 무기한이 될 수도 있다는 얘기를 들어 희망을 가지고 있으며, 미래에 대한 불확실성을 대비하는 느낌이 들기도 한다.

“지금 생각으로는 한 40대 중후반까지 안 쓰더라도 계속 유지를 할 생각이에요. 1년마다 저장해 놓는 저장비용이 있기는 한데 이게 막 크진 않아서... 또 제 몸의 건강이 어떻게 될지 모르는 상황이기 때문에 오픈해 놓은 상황이에요.” (참여자 4)

“한 45살까지는 계속 보관하지 않을까 싶어요.” (참여자 5)

##### 2) 사회적 제약과 개인적인 욕구 사이에서 느끼는 괴리감으로 고민함

참여자는 결혼과 자녀 계획에 대한 고민과 불안감을 안고 있으며, 아이를 갖는 것에 대한 현실적인 어려움을 느끼면서 사회적인 제약과 현실 사이에서 자신만의 선택을 고민하고 있었다.

“결혼은 제 마음대로 안 되니까... 저도 나이가 내년에 결혼을 안 하면 너무 늦긴 하잖아요. (중략) 마음이 급해가지고 아무하고나 결혼할까 봐 난자를 얼리긴 했는데… 병원에서는 난자만 있으면 50살이 되어도 임신이 가능하다고 했지만, 그 나이에 애를 낳고 싶지는 않아요.” (참여자 1)

“우리나라에서 냉동 난자를 사용하려면 결혼을 해야 되잖아요. (중략) 애는 키워보고 싶은데... 이제 제 마음에 들지 않는, 사랑하지 않는 남자랑 애 때문에 결혼할 수는 없잖아요.” (참여자 2)

##### 3) 미사용 시 제도적 틀 내에서의 적절한 사용에 대해 고민함

참여자는 향후 동결보존한 난자를 사용하지 않는 경우에 기증이나 연구용으로 사용될 가능성에 대해 고민하며, 자신의 난자가 실험용으로 사용되는 것에 불편함을 느끼지만 결국 그 난자가 필요할 때 도움이 될 수 있기를 바라는 마음도 가지고 있다.

“일단 계속 가지고 있으면 나중에 어떻게 쓰든지 힘든 사람 나눠주든지 어쩌든지 아무튼 어딘가 쓰겠지요. 그렇게 힘들게 했는데 버릴 수는 없어요 절대.” (참여자 3)

“나중에 기증이라든지 연구용으로 뭘 한다든가 이런 것도 있기는 하던데 마냥 오케이는 아닌 것 같아요. 어떤 제도랑 어떤 규칙 안에서 그게 이루어지는지가 중요할 것 같아요.” (참여자 5)

##### 4) 동결보존한 난자 품질에 대한 신뢰와 불안의 공존

참여자들은 난자동결보존과 관련된 시술을 불확실한 미래에 대한 대비책으로서, 심리적인 안정감을 주는 중요한 선택으로 생각한다. 이들이 삶에서 중요한 결정을 내렸고, 그 과정에서 얻는 자유와 안도감은 미래에 대한 불안을 덜어주었다. 난자동결보존을 통해 결혼이나 인간관계에 대한 부담을 경감시키는 동시에 미래에 대한 희망을 보다 현실적인 방식으로 준비했다고 생각한다.

“난자도 냉동식품같은 거랑 비슷해서 시간이 오래되면 큰 차이는 없긴 하지만 품질이 점점 떨어진다고 해서 추가 채취를 해야 하나 고민이에요.” (참여자 2)

“진짜 든든한 보험 하나 가입해 놓는다는 생각으로...” (참여자 3)

“500만 원짜리 난자 냉동을 딱 해놓고 나니 결혼이나 사람 만나고 할 때 큰 스트레스가 좀 줄어든 것 같아요.” (참여자 4)

“시간이 지나도 임신 성공률이 그러니까 뭔가 이 기술이 개발된 지 엄청 오래되지 않았으니까 이렇게 해 놓으면 수십 퍼센트의 확률로 수정이 된다고 하지만 한 사람이 별로 많이 없으니 거기서 나온 통계자료도 그렇게 믿을 만한 것이 못되지 않을까 두려움이 있어요.” (참여자 5)

#### 주제 6. 선택의 자유와 미래를 위한 주도권 확립이 불투명함을 느낌

##### 1) 국가적 지원의 부족

참여자들은 사회적 지원과 정책적 노력이 더욱 체계적이고 포괄적으로 확장되어야 하며, 출산율 문제를 해결하기 위한 진지한 접근이 필요하다고 생각한다. 특히 일부 지자체에서 지원이 있기는 하지만 저출산 문제를 해결하기 위해서는 실질적인 지원이 필요하다고 의견을 내고 있다.

“노산이다 뭐다 그렇게 말이 많은데 내가 남편이 없다는 이유로 나라에서 이걸 1원도 지원을 안 해주는 게 말이 되냐고요. 나라에서 돈 주고 제발 해달라고 해도 내가 지금 이걸 할까 말까인데 이걸 내가 돈 내고서 이렇게까지 해야 되나 처량해가지고...저는 작년에 했는데 올해 갑자기 몇몇 자치단체에서 지원한다고 뉴스가 뜨는 거예요.” (참여자 3)

“기혼자들에게 육아수당을 주는 것처럼 미혼 여성에 대한 지원이 있어야 한다고 생각해요. 20대에 국가 정책적으로 난자를 동결해주면 사회적인 문제가 많이 해결이 될 것 같은데.” (참여자 4)

##### 2) 미래를 위한 선물

참여자들은 미혼 여성들이 미래의 임신을 대비해 미리 난자 냉동보존을 선택하는 것이 옳다고 여기고 있으며, 그 시기는 빠를수록 좋다고 생각한다.

“지금 미디어가 노산에 대해 엄청나게 얘기하고 있는데… 그 노산의 기준을 여성에게만 부과해서 너무 화가 나는데 (중략) 시간을 되돌릴 순 없으니까, 여성이 임신과 관련한 부담이 좀 줄어 들겠다… 또 나이 들면 몸이 아프게 될 수도 있으니까... 다양한 이유로 적극 추천합니다.” (참여자 3)

“어떤 연예인이 TV에 나와서 그런 말을 했던 것 같아요. 본인 딸이 대학생이 되면 그걸 선물해 주겠다. 저는 그 마음이 참… 저게 정말 정답인 것 같다. 나이가 어리면 어릴수록 나중에 수정 확률이 높아진다고 하니까.” (참여자 4)

#### 미혼 여성의 선택적 난자동결보존 경험의 일반적 구조 기술

미혼 여성의 선택적 난자동결보존 경험은 동결보존 과정에 따른 시간의 흐름과 관련된 상황에 따라 나타나며, 개인적, 사회적, 정책적 측면의 고려가 필요하다. 개인적 측면에서 참여자는 교육 수준 및 기간의 연장과 경력 유지 등으로 결혼이 미뤄지면서 선택적 난자동결보존을 선택하고 임신과 같은 노력으로 이를 실행하고 보관하면서 심리적으로 다소간의 여유가 생겼다. 그러나 사회적 측면에서 참여자의 주변인들은 난자동결보존을 실행하는 사람에 대해 편견을 가지고 있거나 소수의 사람만이 이에 대해 지지를 해주고 있어 자신이 미혼 여성의 난임에 대한 두려움을 이용한 산업에 동참하고 있는 것이 아닌지 갈등을 느끼기도 하였다. 정책적 측면에서 참여자들은 선택적 난자동결에 대한 정확한 정보를 제공해주는 곳이 부족하다 보니 병원을 선택할 때 어려움을 느꼈고, 보관된 난자의 사용 혹은 미사용 시 적절한 처리방법 등에 대한 논의가 필요하다고 보았다. 또한 저출산 문제 해결을 위해서라도 선택적 난자동결보존을 위한 비용 지원이 더 늘어나야 한다고 기술하였다.

## Discussion

본 연구에서 선택적 난자동결보존을 삶의 선택지를 넓히는 혁신적 도구로 여기는 구조와 관련하여, 참여자들은 난자동결보존을 미래를 대비하는 보험으로 생각하고, 전통적인 결혼과 출산의 틀을 벗어나 나이의 압박 없이 원하는 시점에 임신을 할 수 있을 것이라 여겨 조급함을 덜 느끼게 되었다고 보았다. 이러한 결과는 네덜란드 미혼 여성의 사회적 난자동결 경험에 대한 질적 연구[25]와 맥락을 같이 한다. 이 연구에서 네덜란드 미혼 여성이 난자동결보존을 시행하는 가장 큰 동기는 급격한 생식능력의 감소와 남성 파트너의 부재였으며, 향후 아이를 갖고자 하는 욕구를 충족시키는 유일한 방법으로 난자동결보존을 선택하였다. 여성들은 자신의 난자가 냉동고에 보존되어 있다고 생각하면 숨 쉴 공간이 확보된 느낌이 들고, 보험을 들어 둔 것 같으며, 어깨에서 무거운 짐이 내려지는 것 같다고 하며 이를 ‘백업 플랜’으로 인식하였다. 그러나 선행연구에 따르면, 선택적 난자동결보존을 시행하는 여성들은 대체로 건강하며 미래에 모성이 될 수 있는 능력을 위협하는 기존 의학적 문제가 없음에도 불구하고 단지 연령 증가에 따른 생식능력 감소 문제를 해결하기 위해 이를 선택하는 경우가 많았고[26], 선택적 난자동결보존을 통한 향후 임신 성공 가능성 등에 대해 충분히 이해하고 있지 못한 측면이 있었다[10]. 또한 스페인[27]과 이란[28]의 미혼 여성의 난자동결보존에 대한 지식, 태도, 인식 조사에서도 생식능력 감소와 임신의 최적 시기에 대한 지식이 부족한 것으로 나타났다. 이러한 연구 결과를 통해 볼 때, 선택적 난자동결에 대한 지식과 이해가 부족하면 동결 보존된 난자의 활용도도 낮을 수 있으므로, 미혼 여성에게 교육과 개별화된 상담을 제공할 필요가 있을 것이다.

참여자들은 선택적 난자동결보존 시도는 삶의 우선순위의 재조정이 필요한 과정이라고 구조화하였다. 이들은 이 나이쯤 되면 결혼해서 자녀가 여럿 있을 줄 알았는데 자신이 원하는 배우자를 찾는 것이 쉽지 않고, 출산 때문에 일을 놓고 싶지는 않지만 공부나 봉사, 주변의 난임부부를 보며 얻는 간접경험을 통해 출산을 위해서는 삶의 우선순위를 재조정할 필요가 있다고 보았다. 이러한 결과는 여성이 출산 연기를 고려하게 되는 이유 중 하나가 교육이나 직장생활을 우선순위에 두기 때문이며[29], 미국 산부인과 레지던트를 대상으로 한 연구[30]에서 임신을 미루는 이유로 70% 이상이 레지던트 교육 과정을 마치기 위함이고, 50% 이상이 직업과 육아 문제를 꼽한다고 한 연구 결과에 의해 뒷받침된다. 그러나 출산을 30세까지 연기했을 때 영구적 비자발적 무자녀가 될 위험은 6%이지만 40세까지 연기하면 그 위험이 35%로 약 6배 증가하게 되므로[31], 선택적 난자동결보존이 여성에게 출산 가능 기간을 연장할 수 있는 기회를 제공하고 의도치 않게 자녀를 갖지 못할 위험을 줄일 수는 있으나 완전히 없앨 수는 없다[29]. 이러한 측면에서 선택적 난자동결보존을 생식 보험으로 활용하는 것은 상당한 위험이 존재하고 비용이 많이 드는 선택이라고 볼 수 있다. 따라서 이에 대한 산부인과적, 심리사회적, 경제적, 법적, 윤리적 문제 등을 포함한 다학제적 논의가 필요할 것으로 보인다.

참여자들은 선택적 난자동결보존을 수행하면서 전통과 변화의 경계에서 갈등을 경험하였다. 이들의 결정에 대해 성별에 따라 각기 다른 시각이 존재하며, 세대 간 가치관의 차이가 드러났다. 부모는 보수적인 태도를 보였지만, 자녀에게는 또 다른 방식으로 응원을 보냈고, 친구들은 이를 산업적 관점에서 비판적 시선을 보내기도 하였다. 이러한 갈등은 사회적 인식과 개인적 선택 사이의 간극을 상징적으로 나타내는 것으로 보았다. 미국에서 선택적 난자동결보존을 시행한 178명과 선택적 난자동결보존을 시행하지 않은 160명의 총 338명을 상담하여 이에 대한 결정에 후회가 없는지를 결정 후회 척도(Decision Regret Scale)로 측정한 결과, 비보존 군은 보존 군보다 중간–심각한 정도의 결정 후회를 더 많이 하는 것으로 나타났다(50% vs. 13%) [32]. 선택적 난자동결보존 이후 대부분의 여성들은 자신의 선택에 대해 높은 만족도를 표현했지만, 시술과 관련된 적절한 정보를 지원받지 못했거나 주변 사람들의 정서적 지지를 제공받지 못한 경우 생식과 관련된 미래에 대한 높은 수준의 불안, 우울, 외로움, 절망감을 표현하기도 하였다[33]는 점은 본 연구 참여자가 사회적 눈치 혹은 과정 공유에 대한 부담을 느낀 것과 맥락을 같이 한다. 따라서 선택적 난자동결보존을 수행하는 미혼 여성에게 가족이나 가까운 지인의 격려가 필요한 것으로 보인다.

참여자들은 선택적 난자동결보존을 준비 및 실행하면서 몸과 마음의 흔들림 속에서 치열한 정보 탐색과 인내 과정을 경험하였다. 본 연구 참여자들은 난자동결보존을 위해 다양한 매체를 활용하여 적절한 병원을 선택하고자 하였으나 이 과정이 쉽지 않았다고 하였다. 이는 대상자가 다양한 출처를 이용하여 검증된 기관을 찾기를 원하지만 생식능력 보존에 대한 전문 지식이 부족한 대상자는 향후 동결보존된 난자의 높은 임신 성공률을 주장하는 평판 좋은 의학 저널에 게재된 과학적 자료에 쉽게 속을 수 있다고 한 연구 결과[34]와 맥락을 같이 한다. 또한 이렇게 보기 좋아 보이는 자료는 실제로는 선택적 난자동결보존을 수행하는 연령이 높거나 다양한 건강 상태에 있는 여성을 대표하지 못하는 경우가 많으므로[35], 미혼 여성들이 이러한 자료를 해석할 때에는 주의를 기울일 필요가 있겠다. 또한 본 연구 참여자들은 의료인으로부터의 난자동결보존과 관련된 체계적 교육과 정보 지원을 원하고 있으나 이들이 부족함을 느끼고 있었다. 이는 의료인들 또한 이러한 새로운 기술에 대한 정보가 부족한 경우가 많다는 것이 한 이유일 것이다. 또한 미혼 여성은 실제로 나이와 관련된 생식능력 감소를 경험하기 전까지 생식능력 보존의 필요성을 잘 모르는 경우가 많다는 연구 결과와[36], 젊은 여성은 산부인과 방문을 잘 하지 않아 의료 서비스 제공자와 정기적으로 교류가 이루어지지 않는다는 연구 결과[37]로 미루어 볼 때, 한국 사회도 많은 유럽 국가처럼 학교의 성 건강 교육에 생식능력 감소와 관련된 주제를 포함하여 교육할 필요가 있다[38]. 아울러 의료인들은 대상자들과 생식능력 감소와 관련된 대화를 미리 나눔으로써[36], 대상자들이 이에 대해 잘 알고 선택할 수 있도록 해줄 필요가 있겠다. 선택적 난자동결보존 과정에서 나타날 수 있는 신체적 변화는 난소 자극으로 인한 두통, 피로, 오심, 과민성, 유방 압통, 복통, 체중 증가뿐만 아니라 심한 경우 복수 및 흉막 삼출, 호흡곤란, 탈수, 구토, 핍뇨, 혈액 농축, 혈전색전증 등이 있을 수 있다[9,39]. 또한 난자 채취와 관련하여 골반통증, 복강 내 출혈, 골반 감염, 난소 염전 등의 위험이 있으며[10,40], 30개 이상의 난자를 채취할 경우에는 출혈의 위험이 더 높아질 수 있다[41]. 그러므로 의료인들, 특히 생식건강과 관련된 업무를 수행하는 간호사, 의사 및 상담가는 대상자가 선택적 난자동결보존을 결정하기 전에 증거에 기반한 정보(예, 해당 연령대 여성의 선택적 난자동결 성공률)를 충분히 제공하여 정보에 입각한 결정을 내리고, 후회의 여지를 줄이기 위해 포괄적 상담[35]을 제공해 줄 필요가 있겠다.

참여자들은 동결된 난자의 보관과 관련하여 가능성과 제약, 미래를 향한 기대가 얽힘을 경험하였다. 이들은 동결된 난자를 40대 중∙후반까지는 보관하고 싶어하였다. 일부 참여자는 현재 한국에서 미혼 여성이 난자를 해동하여 체외수정 및 배아이식을 받는 것에 법적 제한은 없으나 대한산부인과학회의 윤리지침 등에 의해 의료기관에서 이를 제한할 수 있다는 점을 알고 있었으며, 향후 동결된 난자를 사용하지 않는 경우 이를 어떻게 처리해야 할지에 대해서는 깊게 생각하지 못했지만, 일단 보관을 하고 나니 마음이 든든하다고 하였다. 2016년부터 2022년까지 선택적 난자동결보존을 실시한 여성 167명에 대한 영국의 후향적 연구에서는 이를 수행한 여성의 16%만이 동결된 난자를 사용하였으며[42], 2007년부터 2018년 사이에 선택적 난자동결보존을 실시한 여성 6,362명에 대한 스페인의 다기관 후향적 연구에서는 2.1년 보관 후 사용률은 12.1%에 불과하였다는 선행연구[43]를 통해 볼 때, 사용되지 않은 동결보존된 난자의 처리 문제에 대해서도 고민할 필요가 있을 것으로 보인다. 본 연구 참여자들이 신체적 위험과 재정적 부담을 극복하고 난자동결보존을 선택했지만, 대한산부인과학회의 보조생식술 윤리지침[44]에 따르면 체외수정 수정은 원칙적으로 부부(사실상의 혼인관계에 있는 경우를 포함)관계에서만 시행되어야 한다고 규정하고 있어, 파트너가 없는 미혼 여성은 이를 사용할 수 없다. 이러한 점은 비사용 난자 처리 문제의 해결 필요성을 더욱 강조한다. 선택적 난자동결보존을 허용하는 대부분의 국가에서 동결보존한 난자의 보관기간 관련 규정이 따로 없는 경우가 많다. 난자의 사용 또는 폐기를 결정하는 데에는 5년에서 10년 이상 걸릴 수 있으며[45], 사용하지 않은 난자를 기증할 의향이 50%에서 88% 수준으로 보고되고 있고[46,47], 생식의학 연구의 발전을 위해 난자 기증이 필요하다는 점[28]을 고려할 때, 사용하지 않은 난자의 처리에 대해서도 고민해볼 필요가 있을 것이다.

참여자들은 선택적 난자동결보존과 관련하여, 선택의 자유와 미래를 위한 주도권 확립이 불투명해지는 경험을 하였다. 이들은 우리나라의 출산율을 높이기 위해 미혼 여성에 대한 국가적 지원이 있어야 하며, 홍보 등을 통해 최대한 이른 나이에 이를 시행해야 하고, 대중적 영향에 따라 정보 탐색이나 의사결정이 이루어지지 않도록 정확한 정보적 지원이 있어야 한다고 하였다. 서울시에서는 2023년 9월부터 난자동결보존을 원하는 30–40세 여성에게 최대 200만 원까지 시술 비용을 지원하는 시범사업을 전국 최초로 시작하였고[48] 이후 몇몇 시∙도에서도 시행 중이나, 일반인들은 이를 잘 모르는 경우가 많으므로 지역사회 자치단체에서는 정확한 정보에 기반한 홍보가 필요하다. 난소 예비력 검사나 선택적 난자동결보존에 대한 재정지원을 제공하는 공공정책과 보험 적용 등을 통해 비용의 영향을 완화하면, 정의의 원칙에 따라 사회∙경제적 배경과 관계없이 모든 여성이 정보에 입각하여 자신의 생식능력을 선택할 수 있도록 도울 수 있을 것이다[49]. 40세 이상의 유명인의 임신 성공 및 출산에 대한 언론 보도 등은 40세 이후에도 얼마든지 출산이 가능한 것으로 오해하게 할 수 있다. 이와 같이 미디어가 대중의 인식에 강력한 영향을 미칠 수 있다는 점을 고려하여, 노령 임신의 신체적 위험에 대한 정보도 정확하게 제공될 필요가 있다[36]. 또한 미혼 여성이 난자동결을 희망적인 시술로만 인식하지 않도록 노령 임신의 위험에 대해서도 알려줄 필요가 있겠다[18].

본 연구는 참여자가 모두 전문직 고학력자로만 구성되었다는 점에서 제한점을 가진다. 다양한 배경을 가진 참여자를 모집하기 위해 여러 경로를 통해 대상자 모집을 시도하였으나, 난자동결보존 경험 자체의 노출을 원치 않는 경우가 많아 어려움이 있었다. 향후 다양한 배경을 가진 참여자를 포함한 연구가 필요할 것으로 보인다.

또한 본 연구에서는 현상학적 접근을 바탕으로 참여자의 경험을 심층적으로 탐색하고자 하였으나, 면담 초기 단계에서 연구자가 제시한 비구조화된 질문이 비교적 구체적으로 구성되어 있어 참여자의 자유로운 진술을 제한했을 가능성이 있다. 면담은 1–2회로 계획되었으나, 일부 참여자의 경우 후속 면담을 통해 초기 질문 범위를 넘어서는 경험이 자연스럽게 도출되기도 하였다. 이는 현상학적 방법의 유연성과 심층성을 반영하는 긍정적인 결과이기도 하나, 사전에 설정된 질문들이 연구자의 인식을 일정 부분 투영한 한계로 작용할 수 있음을 고려해야 한다.

본 연구는 기술적 현상학적 방법을 적용하여, 미혼 여성의 시각에서 선택적 난자동결보존을 수행하는 과정에서의 경험을 심층적으로 탐색하고 그 의미와 본질을 이해하고자 하였다. 참여자들은 선택적 난자동결보존을 삶의 선택지를 넓히는 혁신적 도구로 인식하였으며, 이 과정은 삶의 우선순위 재조정, 전통과 변화의 경계에서의 갈등, 몸과 마음의 흔들림 속에서의 정보 탐색과 인내, 가능성과 제약이 얽힌 미래에 대한 기대, 선택의 자유와 주도권 확립의 불투명함 등으로 구조화되었다. 이러한 경험은 생식 자율성을 성취해 나가는 복합적 여정으로 나타났다. 참여자들은 임신 준비에 버금가는 노력과 높은 장벽에도 불구하고 난자동결보존을 수행하였으며, 신체적∙정서적 부담 외에도 제한된 사회적 지지와 정보 부족의 어려움도 함께 경험하였다. 이에 따라 임상 실무에서는 산부인과 간호사와 같은 의료인이 근거에 기반한 정확한 정보를 제공하고 포괄적인 상담을 통해 대상자의 충분한 이해와 결정을 도울 필요가 있다. 나아가 정책적 차원에서는 시술 전 상담 프로토콜의 표준화, 비용 지원 확대, 생식건강 교육 강화 등의 제도적 기반 마련이 요구되며, 향후에는 다양한 사회∙경제적 배경을 지닌 여성을 포함한 후속 연구를 통해 보다 실효성 있는 생식능력 보존 전략이 마련되어야 할 것이다.

## Figures and Tables

**Table 1.** Main interview questions and probes on elective oocyte cryopreservation

Main question	Probing question
1. What do you know and think about elective oocyte cryopreservation?	- How did you first learn about elective oocyte cryopreservation?
	- What sources of information influenced your perception the most?
2. What are your motivations for undergoing elective oocyte cryopreservation?	- Was there a specific event that led you to make this decision?
	- How did external factors (family, career, social norms) influence your motivation?
3. How did you prepare for elective oocyte cryopreservation?	- What steps did you take before undergoing the procedure?
	- What challenges did you face during preparation?
4. What do you think about the costs associated with elective oocyte cryopreservation?	- Did financial factors affect your decision to proceed with or delay the procedure?
	- Do you think there should be financial support for elective oocyte cryopreservation?
5. What were your physical and emotional responses to the elective oocyte cryopreservation process?	- What specific physical symptoms did you experience during the process?
	- How did you manage the emotional challenges associated with the procedure?
6. What do you think about the results of elective oocyte cryopreservation?	- Are you satisfied with the outcome? Why or why not?
	- What concerns do you have about the future use of your stored oocytes?
7. What are your plans for stored oocytes and their future use?	- Have you thought about how long you plan to store your oocytes?
	- What factors will influence your decision to use or discard them?
8. What are your future hopes and plans regarding motherhood?	- How does elective oocyte cryopreservation fit into your long-term life goals?
	- Do you feel pressure from society regarding motherhood?
9. Finally, what are your overall thoughts on elective oocyte cryopreservation, and would you recommend it to others?	- What advice would you give to someone considering elective oocyte cryopreservation?
	- If you could go back, would you make the same decision? Why or why not?

**Table 2.** General and oocyte cryopreservation-related characteristics of interviewees (N=5)

Participant	Age (year)	Education level	Occupation	Menarche age (year)	Number of frozen oocytes	Cost of oocyte cryopresevation (KRW)^*^
P1	42	Graduate school	White-collar worker	10–12	10	4 million
P2	35	Graduate school	White-collar worker	10	30	4 million
P3	33	Graduate school	Business owner	12–13	15	3.5 million to 4 million
P4	35	Graduate school	White-collar worker	8–9	4	5 million
P5	36	Graduate school	White-collar worker	12	23	3.5 million

* KRW: Korean won (1 million KRW is approximately 690 USD).

**Table 3.** Themes and subthemes for elective oocyte cryopreservation in unmarried women

Theme	Subtheme
Innovative tool that expands life choices	· Insurance for the future
	· Action that breaks the traditional framework of marriage and childbirth
	· Expansion of women’s physical freedom and choice
Process that requires readjusting life priorities	· Postponed marriage
	· Conflict between social expectations and personal goals
	· Responsibilities and concerns as future parents
Conflict felt at the boundary between tradition and change	· Treated as a new woman, a strong woman
	· Feeling ambivalent at the crossroads of values ​​between generations
	· Feeling skeptical about the gap between industrialized procedures and social perception
Fierce information search and perseverance amidst the turmoil of body and mind	· Putting in the effort to prepare for pregnancy
	· Making an effort to choose a hospital using various media
	· Experiencing persistent emotional fluctuations and psychological distress caused by side effects of hormone injections
	· Enduring the challenge of physical changes and difficulties in recovery
Intertwined with possibilities, constraints, and expectations for the future	· Establishing a mid- to long-term maintenance plan
	· Struggling with the dissonance between social constraints and personal desires
	· Thinking about appropriate use within the institutional framework
	· Coexistence of trust and anxiety about the quality of frozen oocytes
Feeling uncertain about freedom of choice and taking initiative for the future	· Lack of national support
	· Gift for the future

**Table 4.** Examples of theme-subtheme-meaning unit analysis

Theme	Subtheme	Meaning unit
Innovative tool that expands life choices	Insurance for the future	· Buying time
		· Keeping insurance in case I cannot give birth right away
	Expansion of women’s physical freedom and choice	· Given the option to choose the age at which you would like to become pregnant
		· If I get the chance, I would like to have a child and raise him/her
Process that requires readjusting life priorities	Conflict between social expectations and personal goals	· I don’t want to live according to the schedule society wants
		· I don’t want to lose my job because of having a baby
	Postponed marriage	· Having a hard time finding the partner I want
		· I was shocked to learn that starting at age 35, pregnancies are considered high-risk
